# APOBEC4 Enhances the Replication of HIV-1

**DOI:** 10.1371/journal.pone.0155422

**Published:** 2016-06-01

**Authors:** Daniela Marino, Mario Perković, Anika Hain, Ananda A. Jaguva Vasudevan, Henning Hofmann, Kay-Martin Hanschmann, Michael D. Mühlebach, Gerald G. Schumann, Renate König, Klaus Cichutek, Dieter Häussinger, Carsten Münk

**Affiliations:** 1 Clinic for Gastroenterology, Hepatology, and Infectiology, Medical Faculty, Heinrich-Heine-University Düsseldorf, Düsseldorf, Germany; 2 Division of Medical Biotechnology, Paul-Ehrlich-Institute, Langen, Germany; 3 Biostatistics, Paul-Ehrlich-Institute, Langen, Germany; 4 Product Testing of Immunological Medicinal Products for Veterinary Uses, Paul-Ehrlich-Institute, Langen, Germany; 5 Host-Pathogen Interactions, Paul-Ehrlich-Institute, Langen, Germany; 6 Sanford Burnham Prebys Medical Discovery Institute, Immunity and Pathogenesis Program, La Jolla, California, United States of America; Institut de Recherches Cliniques de Montréal (IRCM), CANADA

## Abstract

APOBEC4 (A4) is a member of the AID/APOBEC family of cytidine deaminases. In this study we found a high mRNA expression of A4 in human testis. In contrast, there were only low levels of A4 mRNA detectable in 293T, HeLa, Jurkat or A3.01 cells. Ectopic expression of A4 in HeLa cells resulted in mostly cytoplasmic localization of the protein. To test whether A4 has antiviral activity similar to that of proteins of the APOBEC3 (A3) subfamily, A4 was co-expressed in 293T cells with wild type HIV-1 and HIV-1 luciferase reporter viruses. We found that A4 did not inhibit the replication of HIV-1 but instead enhanced the production of HIV-1 in a dose-dependent manner and seemed to act on the viral LTR. A4 did not show detectable cytidine deamination activity *in vitro* and weakly interacted with single-stranded DNA. The presence of A4 in virus producer cells enhanced HIV-1 replication by transiently transfected A4 or stably expressed A4 in HIV-susceptible cells. APOBEC4 was capable of similarly enhancing transcription from a broad spectrum of promoters, regardless of whether they were viral or mammalian. We hypothesize that A4 may have a natural role in modulating host promoters or endogenous LTR promoters.

## Introduction

The AID/APOBEC (apolipoprotein B mRNA-editing enzyme, catalytic polypeptide-like) polynucleotide (deoxy) cytidine deaminases family consists of AICDA (activation-induced cytidine deaminase, AID), APOBEC1 (A1), APOBEC2 (A2), APOBEC3 (A3), which has the following seven paralogues in humans: A3A–A3D, A3F–A3H, and APOBEC4 (A4) [1–5]. These enzymes have a diverse range of functions and substrate specificities. Cytidine deamination of single-stranded DNA or RNA was shown to be the principal activity of the AID, A1, and A3 proteins in biochemical and cell culture assays, but such evidence is lacking for A2 and A4 proteins.

Cytidine deaminases of the A3 gene family can inhibit long terminal repeat (LTR)—and non-LTR-retrotransposons and have broad antiviral activity against retroviruses such as HIV and murine leukemia virus (MLV), hepadnaviruses, and non-related viruses [6–21]. A3s mainly act by deaminating cytidine into uridine using single-stranded DNA as a substrate (for review, see [22]). DNA editing introduces hypermutations of the viral genome that eventually render the target genome inactive. Conversely, retroviruses have evolved countermeasures to prevent encapsidation of A3s into viral particles. For example, the Vif protein in lentiviruses, the Bet protein in foamyviruses, the glycosylated Gag (glyco-Gag) protein in MLV, and the nucleocapsid protein in Human T-cell lymphotropic virus accomplish this counteraction using different mechanisms [17, 19, 20, 22–28].

AID is a B lymphoid protein that deaminates chromosomal DNA, thereby inducing somatic hypermutations and gene conversion. Furthermore, AID stimulates class switch recombination in B cells [29–35]. AID can restrict LINE-1 (L1) retrotransposition [15, 36, 37], but it is inactive against HIV-1 [38–40]. A1 catalyzes the cytidine-to-uridine editing of apolipoprotein B mRNA in the intestine [41, 42]. Editing generates a premature stop codon, which is translated to produce a truncated form of apolipoprotein B protein, termed *apoB48*, that has distinct functions in lipid transport [43]. The editing mechanism is highly specific for residue C6666 and works in conjunction with A1 complementation factor [44]. Other mRNA targets for A1 editing were recently identified [45]. A1s of rabbit and rodents inhibit both MLV and HIV-1 by mutating the viral RNA and DNA; in contrast human A1 does not edit *in vitro* [39, 46–49]. In addition, L1 retrotransposons can be restricted by A1s derived from rodents and rabbits, but this effect is weak for human A1 [15, 50]. A2 plays an important role in regulating and maintaining muscle development in mammals [51]. A2 did not exhibit cytidine deaminase activity of DNA substrates in bacterial or yeast mutation assays [52, 53]. Human A2 lacks inhibitory activity against retrotransposons [9, 54, 55] and HIV-1 [38, 40], and murine A2 does not inhibit or edit MLV [46].

A4 protein is more closely related to A1 than to the other APOBECs, and the A4 gene is conserved in chimpanzee, rhesus monkey, dog, cow, mouse, rat, chicken, and frog [3]. A4 is considered to be a putative cytidine-to-uridine editing enzyme. However, experiments conducted using A4 overexpression in yeast and bacteria failed to show cytidine deamination activity in DNA [52]. In mice, the A4 gene is expressed primarily in testis [3], which suggests that it may be involved in spermatogenesis. Whether human A4 participates in intrinsic immunity against HIV as demonstrated for A3s and A1 is unknown, but these anti-viral activities of its sister proteins suggest that it might be possible. Therefore we set out to evaluate the effect of human A4 on the replication of HIV-1 *in vitro*.

## Results

### Analysis of A4 expression in cell lines and human testis tissue

**Fig 1:**
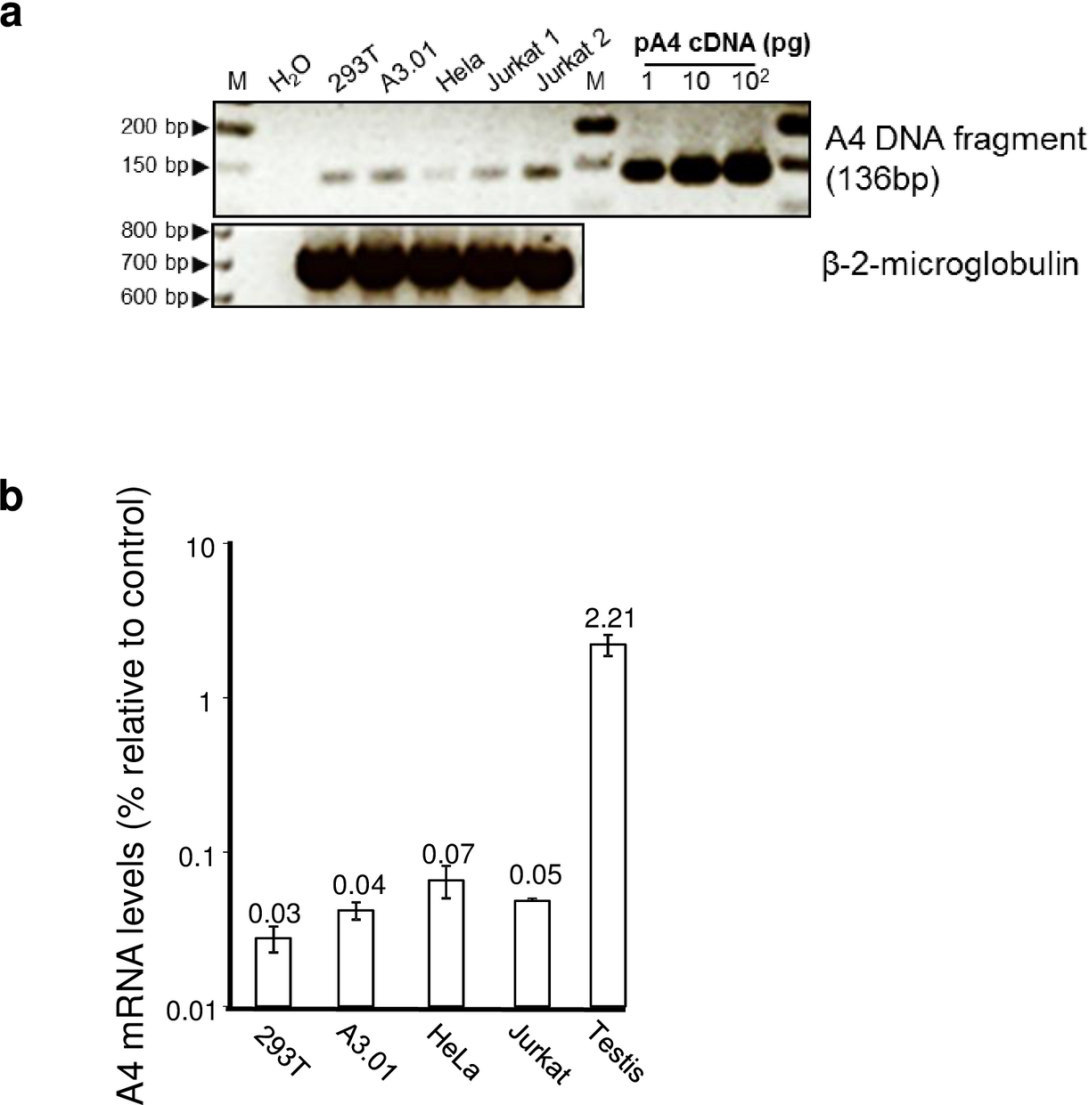
Differential expression of A4. (a) A4 expression was determined by semi-quantitative RT-PCR. Low level A4 amplification by PCR using equal amount of cDNA prepared from total RNA of 293T, A3.01, Hela, and Jurkat cell lines. As a control, β-2-microglobulin (ß-2-M) cDNA was amplified. Water instead of template served as a background control and a plasmid coding for A4 cDNA (pA4 cDNA) served as a positive control. M: 50 bp DNA ladder. (b) Levels of A4 expression were determined by quantitative real-time RT-PCR and measured relative to endogenous HPRT1 RNA levels. A4 is expressed at a high level in human testis tissue, while 293T, HeLa, A3.01 and Jurkat cells exhibit very low A4 expression. Error bars indicate standard deviation.

**Fig 2:**
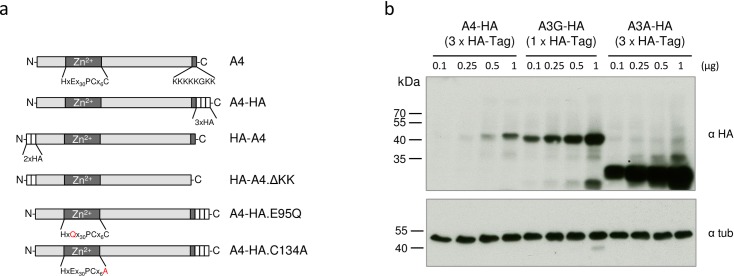
Expression of the A4-HA fusion proteins. (a) Schematic representation of protein domains and motifs found in the human A4 protein and tested variants. Zn^2+^: presumed zinc-binding domain. HA (white boxes): HA-tag. KKKKKGKK: polylysine domain. (b) Increasing amounts of A4-HA (3xHA-tags), A3G-HA (1xHA-tag) and A3A-HA (3xHA-tags) expression plasmids were transfected into 293T cells followed by immunoblot analysis of the transfected cells using an anti-HA antibody. Immunoblot analysis with anti-tubulin (tub) antibody served as loading control. α, anti.

**Fig 3:**
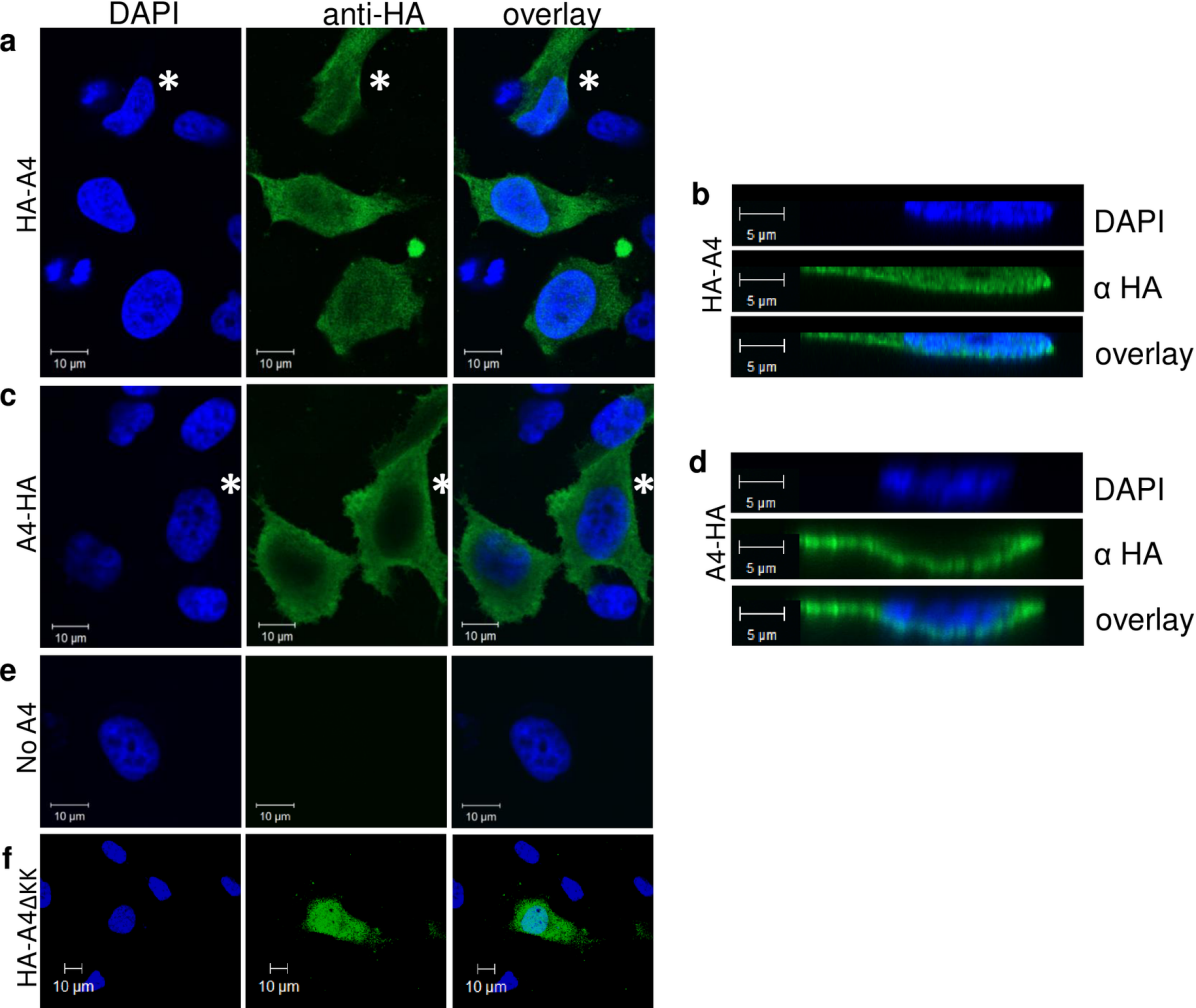
Subcellular localization of A4 in transfected cells. Immunofluorescence confocal laser scanning microscopy images of HeLa cells transfected with N- or C-terminal HA-tagged A4 (HA-A4 and A4-HA). (a, b) HA-A4 proteins show cytoplasmic and nuclear localization. (c, d) A4-HA proteins show cytoplasmic localization. (a, c, e, f) x-y optical sections. (b, d) x-z vertical scanning image of indicated cells (see asterisks). (e) Mock transfected cells (no A4). (f) HA-A4ΔKK transfected cells show cytoplasmic and nuclear localization. To detect A4 (green) immunofluorescence, cells were stained with an anti-HA antibody. Nuclei (blue) were visualized by DAPI staining. α, anti.

Based on information of public repositories (e.g. GenBank) A4 is detectable mainly in human testis, and neither full length A4 mRNAs nor expressed sequence tags (ESTs) have been identified in blood cells, lymphoid tissues, T cells or macrophages. To functionally test A4 in cell culture, we first wanted to determine whether widely used human cell lines express A4. To this end, semi-quantitative RT PCRs on total RNA from the 293T, HeLa, A3.01 T, and Jurkat T cell lines were conducted and the weakly detected PCR products were cloned and sequence verified (Fig 1A). We further compared the A4 expression levels of these cell lines to A4 expression in human testis tissue by quantitative real-time RT PCR on total RNA. Data demonstrate that A4 expression levels in testis are approximately 30- to 50-fold higher than those in the tested cell lines (Fig 1B). A4 expression plasmids were generated with either N-terminal or C-terminal HA-tags or without any tag (HA-A4, A4-HA, A4, Fig 2A). A4 constructs expressed 10- to 100-fold less protein than A3 plasmids expressed from the same vector as shown for A4-HA (3xHA-tag) in comparison with A3G-HA (1xHA-tag) and A3A-HA (3xHA-tag) (Fig 2B). To study the subcellular localization of the differently tagged A4 proteins, we analyzed the expression of these proteins in transfected HeLa cells using confocal microscopy. HA-A4 was localized in both cytoplasm and nucleus (Fig 3A and 3B). A4-HA exhibited a predominantly cytoplasmic distribution (Fig 3C and 3D). Untagged A4 could not be detected, because there was no A4-specific antibody available. To analyze if the characteristic polylysine stretch (KKKKKGKK) at the C-terminus is important for nuclear localization of A4, an N-terminal HA-tagged mutant lacking the polylysine domain (HA-A4ΔKK, Fig 2A) was tested. Only few cells showed expression of this protein, however, if expressed, HA-A4ΔKK was detectable in nucleus and cytoplasm, suggesting that the polylysine stretch does not function as a nuclear localization motif (Fig 3F).

### A4 expression results in an increased HIV-1 particle yield

**Fig 4:**
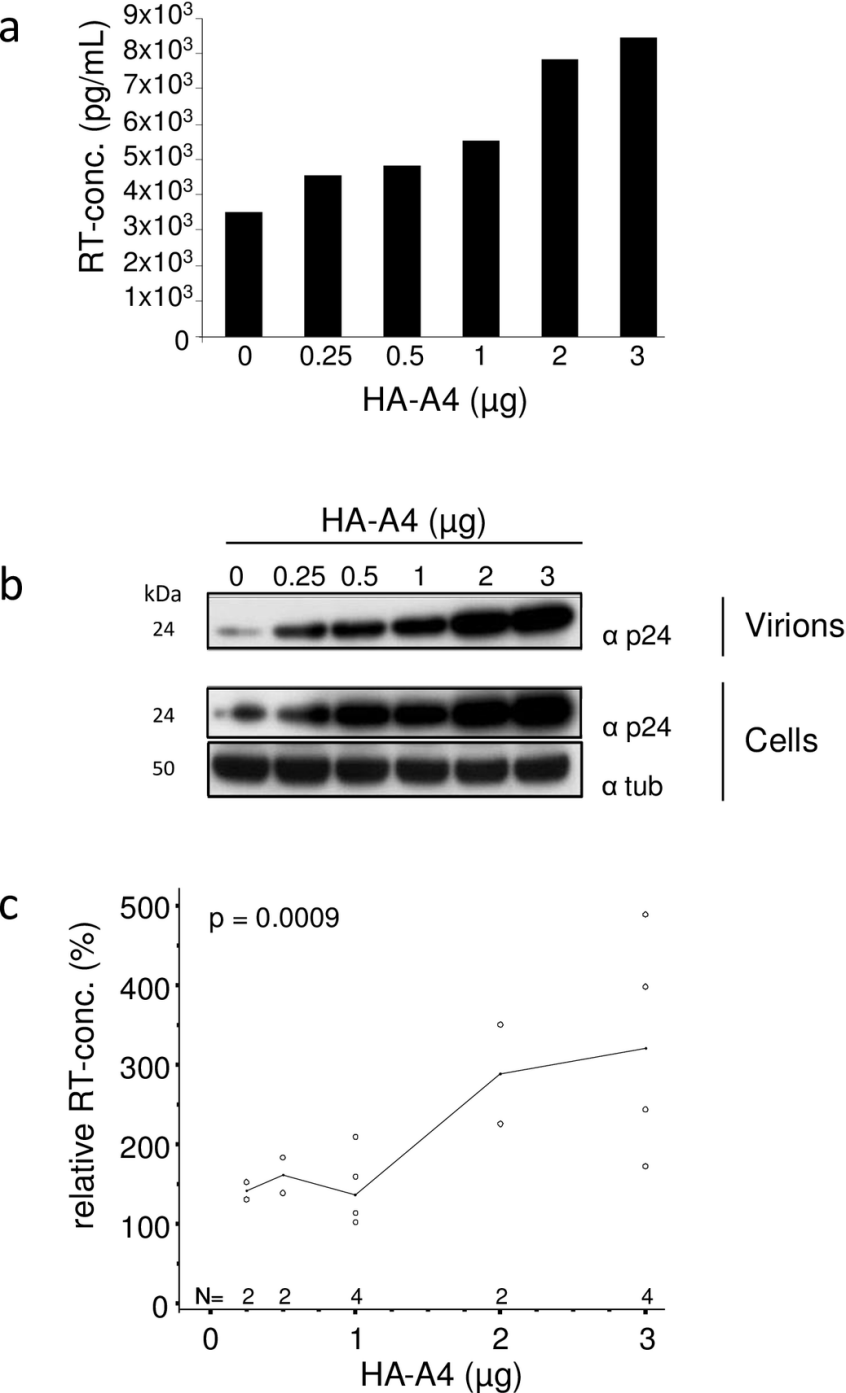
A4 enhances the expression of HIV-1. (a) HIV-1 genome expression plasmid was co-transfected with increasing amounts of HA-A4 expression plasmid, as indicated. A4 increases the production of HIV-1 particles as measured by the RT activity in the supernatant of the transfected cells. (b) Immunoblot analysis of virions and transfected 293T cells (same cells as in (a)). Immunoblots of virions and cell lysates were probed with anti-p24 (capsid) antibody. Anti-tubulin (tub) antibody served as loading control. α, anti. (c) RT concentrations in the supernatant of cells co-transfected with HA-A4 and HIV-1 plasmids relative to supernatant of cells co-transfected with empty vector and HIV-1, as in (a), summary of four independent experiments, median indicated. Evaluation of RT activity data was performed by means of a multifactorial analysis of variance (ANOVA).

To determine the effect of A4 on HIV-1 particle production, we co-transfected increasing amounts of the HA-A4 expression plasmid with a constant amount of HIV-1 expression plasmid (pNL4-3 [56]). The total amount of transfected DNA was kept constant by replacing HA-A4 with the empty expression plasmid (pcDNA3.1zeo). Two days post transfection, we quantified virus production by measuring viral reverse transcriptase (RT) activity in the cell culture supernatant (Fig 4A) and tested the cell lysate for expression of HIV-1 Gag (p24) by immunoblot analysis (Fig 4B). Transfection of incremental amounts of HA-A4 plasmid caused a 2.5-fold increase in the amount of released viral particles as reflected by the RT activity detected in cell culture supernatants. Immunoblot analyses of viral lysates concentrated from the cell culture supernatant also demonstrated the A4 stimulating effect on virus expression (Fig 4B). This positive effect of HA-A4 on late stage HIV-1 particle production was highly reproducible, as demonstrated by data from four independent experiments (Fig 4C). These results were consistent with experimental findings using untagged A4 protein (data not shown).

### Production but not infectivity of HIV-1-luciferase is enhanced by A4 expression

**Fig 5:**
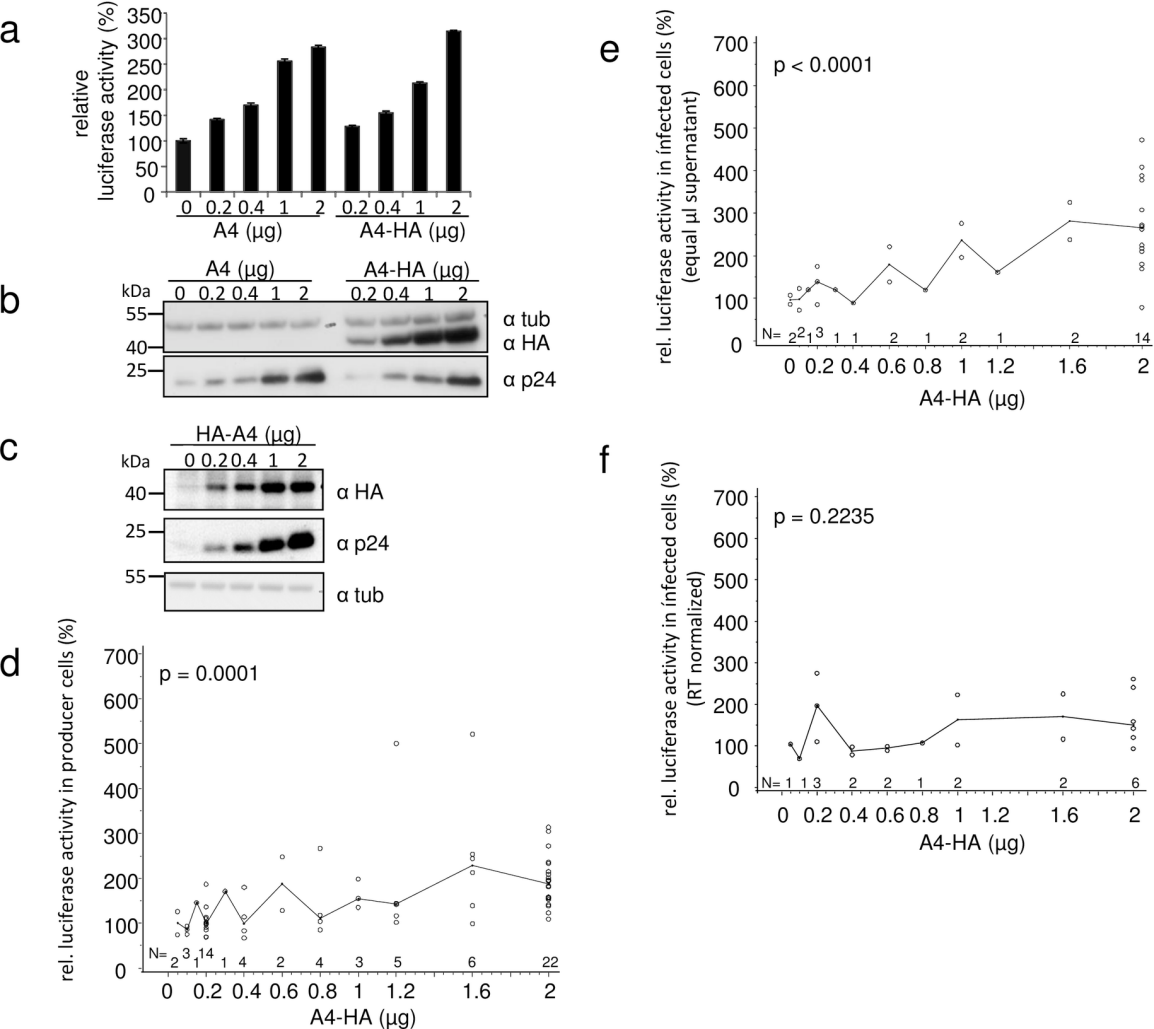
Presence of A4 does not affect HIV-1 infectivity. HIV-1 reporter virus NL-Luc R^-^E^-^ (VSV-G) was produced in 293T cells in the presence of increasing amounts of A4 (no tag) and A4-HA (C-terminal HA-tag). A4 and A4-HA increase in a dose-dependent manner both (a) the virus-encoded luciferase activity and (b) the expression of intracellular viral capsid (p24) in the transfected virus producing cells as demonstrated by immunoblot analysis (same cell lysates used in (a) and (b)). Error bars indicate standard deviation. (c) Immunoblot analysis of intracellular viral p24 (capsid) expression. Similar as in (a) and (b), NL-Luc R^-^E^-^/VSV-G was co-transfected with increasing amounts of HA-A4 plasmid (N-terminal HA-tag), as indicated. Immunoblots of cells were probed with anti-p24 (capsid) antibody. A4-HA expression in transfected cells was detected by immunoblotting using anti-HA antibody. Anti-tubulin (tub) antibody served as loading control. α, anti. (d) Relative viral luciferase activity in cells co-transfected with A4-HA and HIV-1 plasmids, as in (a). Summary of 28 independent experiments, median indicated. A4-HA was transfected in increasing amounts. (e) Equal volumes of supernatants of cells co-transfected with NL-Luc R^-^E^-^/VSV-G and increasing amounts of A4-HA were used to infect HOS cells. Intracellular luciferase activities were determined in infected cells; summary of 16 experiments (a subset of the experiments shown in (d)), median is indicated. (f) A subset of samples (seven experiments) used in (e) was quantified for RT concentrations. RT normalized supernatants of cells co-transfected with NL-Luc R^-^E^-^/VSV-G and increasing amounts of A4-HA were used to infect HOS cells. Intracellular luciferase activities determined in infected cells, median is indicated. (d—f) Statistical evaluation of reporter luciferase activity data was performed by means of a multifactorial ANOVA.

We used VSV-G pseudotyped HIV-1 luciferase virus (NL.Luc R^-^E^-^ [57]) to test whether increasing the levels of expressed A4 influences HIV-1 production and infectivity. Co-transfection of A4 expression plasmids (data are shown for A4 and A4-HA plasmids in Fig 5A) and NL-Luc resulted in a dose-dependent increase of intracellular virus-encoded luciferase activity in transfected 293T cells. Presence or absence of the viral Vif protein (using the *vif*-deficient NL.Luc R^-^E^-^Δvif/VSV-G in the same set of experiments) had no detectable effect on the A4-induced stimulation of NL.Luc (data not shown). Immunoblot analysis of lysates isolated from the transfected cells confirmed the A4 dose-dependent expression of viral capsid p24 (Fig 5B). Fig 5C shows that co-expression of HA-A4 with NL.Luc also caused a similar boost of Gag expression, indicating that the location of the HA tag did not influence virus production enhancement by A4. Results from 28, 16, and seven independent experiments using different amounts of the A4-HA plasmid together with NL.Luc are summarized in Fig 5D, 5E and 5F, respectively. These results confirmed a significant increase in NL-Luc-mediated luciferase activity in the transfected virus producer cells (Fig 5D). When testing equal volumes of cell culture supernatants for the presence of infectious HIV reporter virions, we also detected a dose-dependent increase in luciferase activity in infected cells (Fig 5E). However, when equal concentrations of viral particles normalized for RT activity were used escalating levels of A4-HA did not cause a significant increase in infectivity (Fig 5F). The summarized individual experiments did not always cover all ranges of applied plasmid concentrations (Fig 5D), and single virus samples obtained from a subset of experiments were used to study particle infectivity (Fig 5E and 5F). Taken together, these data indicate that A4 expression enhances the production of HIV-1, but does not change its infectivity.

### Stable A4 expression enhances multiple-cycle replication of HIV-1

**Fig 6:**
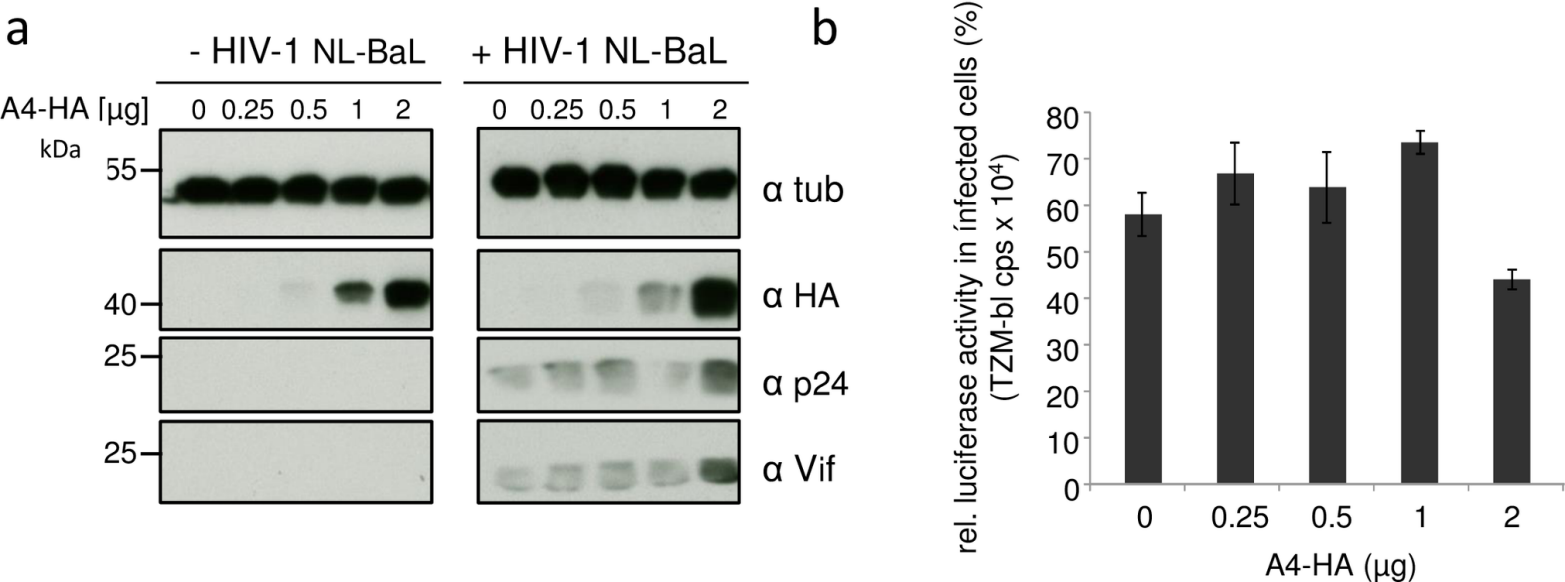
A4 enhances expression of CCR5-tropic HIV-1. (a) Increasing amounts of A4-HA expression plasmid were co-transfected with HIV-1 NL-BaL and immunoblot analysis of co-transfected 293T cells were performed. Immunoblots were probed with anti-p24 (capsid), anti-Vif, anti-HA and anti-tubulin (tub) antibodies. α, anti. (b) Infectivity of RT-normalized viral supernatant of the transfected cells from (a) were used to infect TZM-bl luciferase reporter cells. cps, counts per second. Data are represented as the mean with SD. Statistically significant differences between no A4 and A4 groups were analyzed using the unpaired Student’s t-test with GraphPad Prism version 5 (GraphPad software, San Diego, CA, USA). Validity of the null hypothesis was verified with significance level at α value = 0.05. NS: not significant.

To test whether A4 can also enhance production of CCR5-tropic HIV-1, we co-transfected the replication competent HIV-1 NL-BaL plasmid [58] with A4 expression plasmid and measured the infectivity of RT value normalized particles using the HIV-reporter cell line TZM-bl [59]. The expression of A4-HA resulted in enhanced expression of NL-BaL, as demonstrated by immunoblots probed for viral capsid p24 and Vif proteins (Fig 6A). The viral particles harvested from this experiment demonstrated similar infectivity after normalization for RT activity (Fig 6B).

**Fig 7:**
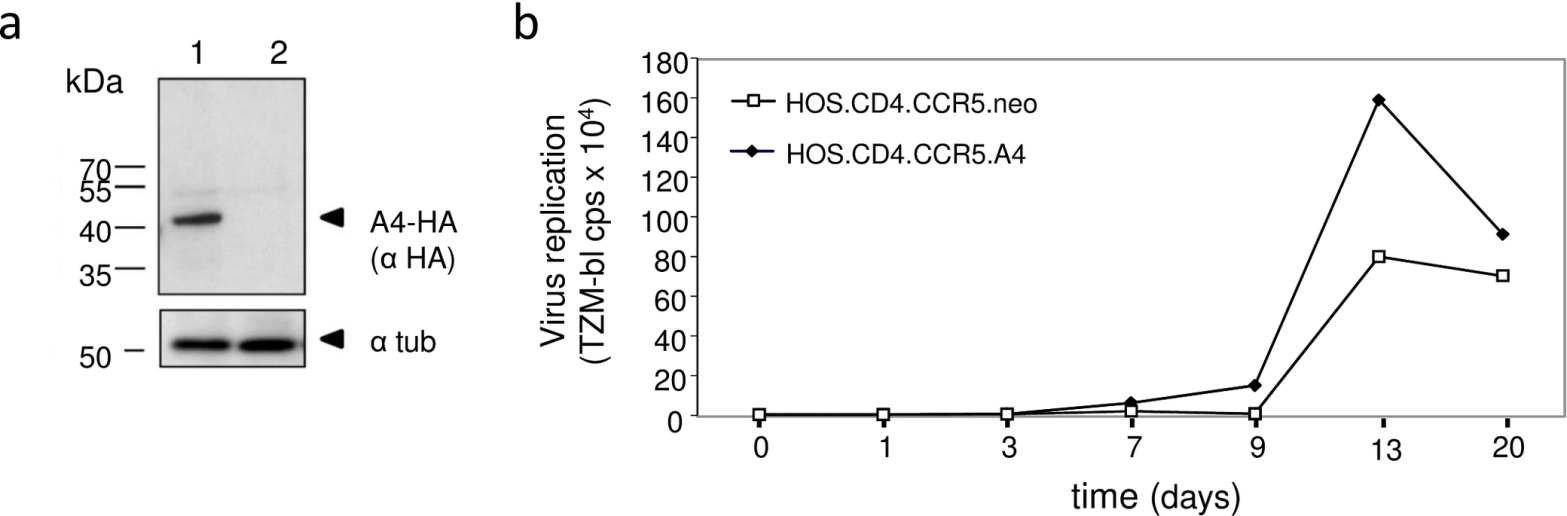
A4 enhances multiple cycle replication of HIV-1. (a) Immunoblot analysis of A4-HA expressing HOS.CD4.CCR5.A4 cells (1) and empty retroviral vector just encoding G418-resistance containing HOS.CD4.CCR5.neo cells (2) using an anti-HA antibody. Cell lysates were also analyzed for equal amounts of total proteins by using anti-tubulin antibody. (b) HOS.CD4.CCR5.A4 and HOS.CD4.CCR5.neo cells were infected with HIV-1 clone NL-BaL, MOI of 0.01. Virus replication was monitored by testing the cell supernatants on TZM-bl cells and measuring luciferase activity.

To analyze whether A4 expression can also enhance spreading replication of HIV-1, we generated a stable A4-HA expressing cell line derived from HOS.CD4.CCR5 cells [60] using a G418-selectable retroviral A4-expressing vector (Fig 7A). As a control, we generated HOS.CD4.CCR5.neo cells transduced with a retroviral vector just encoding the G418-resistance gene. The cell lines were infected with NL-BaL, and virus spread was monitored for 20 days (Fig 7B). HIV-1 showed comparable overall virus replication kinetics in both cell lines; however, HIV-1 replicated in the A4 expressing cells more efficiently resulting in 2–3 fold increased virus titers. These data are consistent with our finding that A4 stimulated HIV expression in the transient transfection experiments, supporting the premise that A4 modulates HIV-1 replication.

### HIV enhancement is not mediated by cytidine deamination

**Fig 8:**
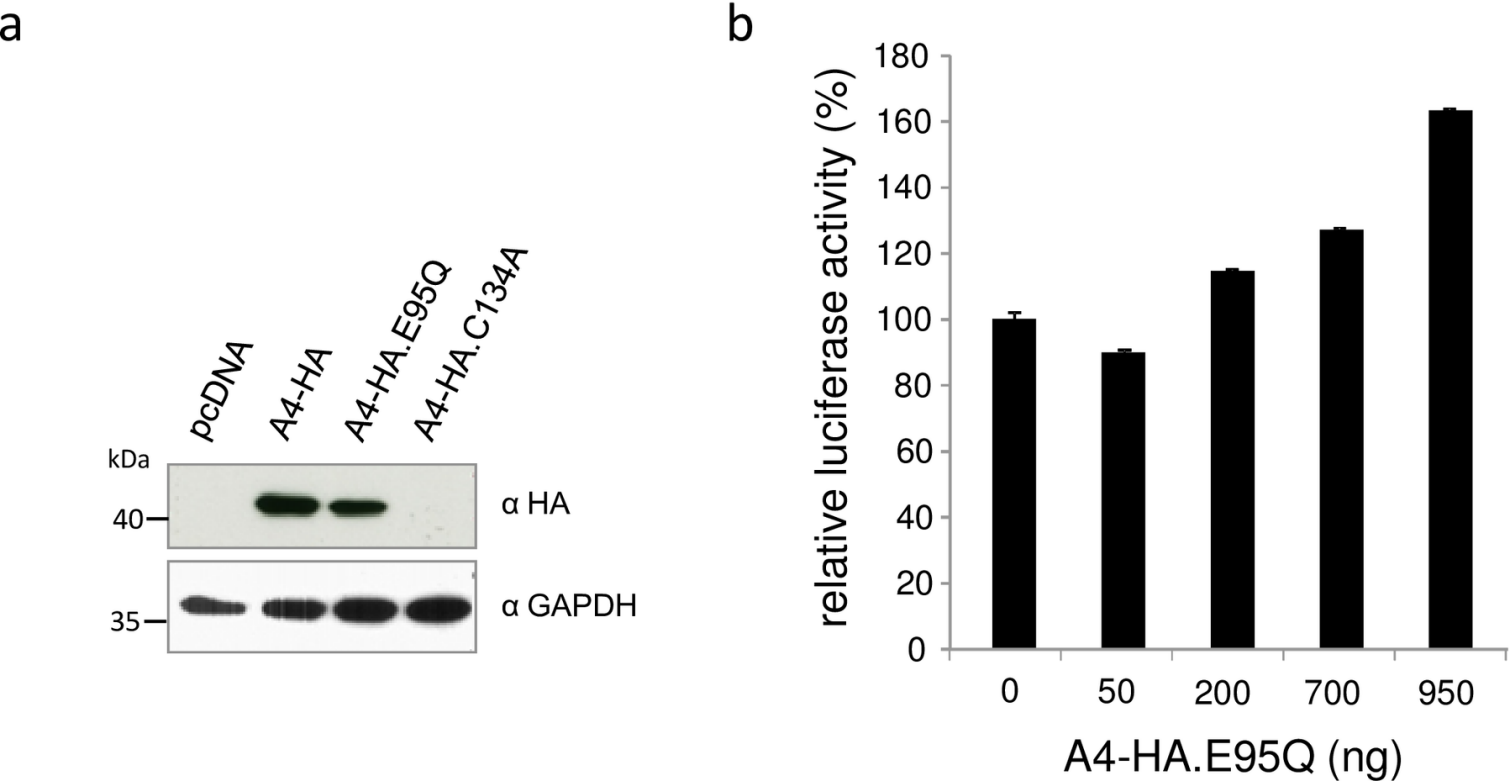
Active site mutation has no influence on A4 activity. (a) Protein expression of A4-HA, A4-HA.E95Q and A4-HA.C134A detected by anti-HA immuno blot analysis, showing equal amounts of A4-HA and A4-HA.E95Q, but lack of A4-HA.C134A expression in transfected cells. (b) HIV-1 reporter virus (NL-Luc R^-^E^-^) was co-transfected with increasing amounts of expression plasmid for A4-HA.E95Q. Virus encoded luciferase activity in the transfected cells was enhanced by A4-HA.E95Q in a dose-dependent manner.

To test whether cytidine deamination activity is associated with the described A4 effect, we generated the active site mutants E95Q and C134A, in which the zinc-coordinating motif HxEx_30_PCx_6_C (E95 and C134 underlined, x can be any amino acid) was mutated (Fig 2A). Unexpectedly, only the A4-E95Q construct expressed detectable protein, precluding A4-C134A mutant from functional studies (Fig 8A). To analyze if the active site mutation has any effect on virus production, HIV-1 luciferase plasmid (NL.Luc R^-^E^-^) was co-transfected with increasing amounts of A4-HA.E95Q expression plasmid and luciferase activity was measured in virus producer cells (Fig 8B). 293T cells showed higher virus-encoded luciferase activity after transfection of A4-HA.E95Q in a dose-dependent manner, comparable with the luciferase enhancement after transfection of wildtype A4 (Fig 5A), indicating that cytidine deamination activity of A4 protein is dispensable for the described HIV enhancing effect.

### A4 lacks detectable cytidine deaminase activity

**Fig 9:**
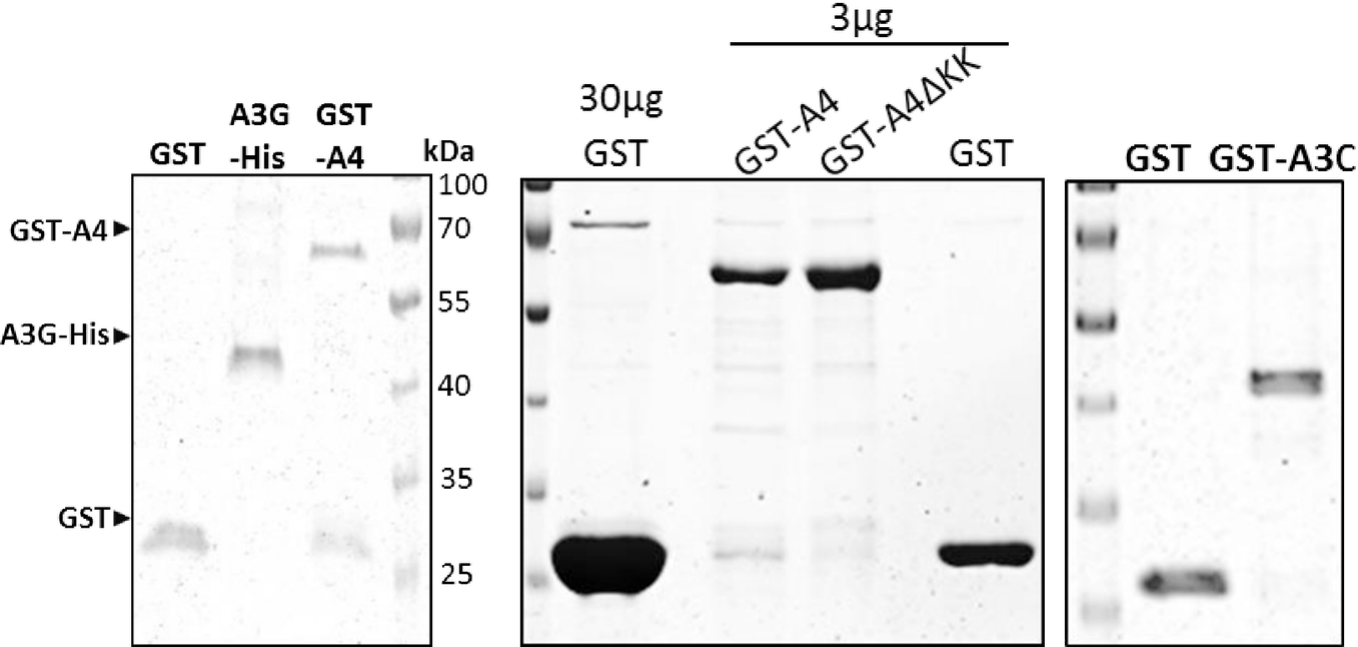
Recombinantly produced and affinity purified *E*. *coli*-derived GST, GST-A3C, GST-A4, GST-A4-ΔKK proteins and 293T cell-derived A3G-His protein were resolved on a 10% SDS gel. Purity of the proteins was determined by staining the gel with Coomassie blue. GST-A4, A3G-His and GST proteins are indicated according to their molecular mass.

**Fig 10:**
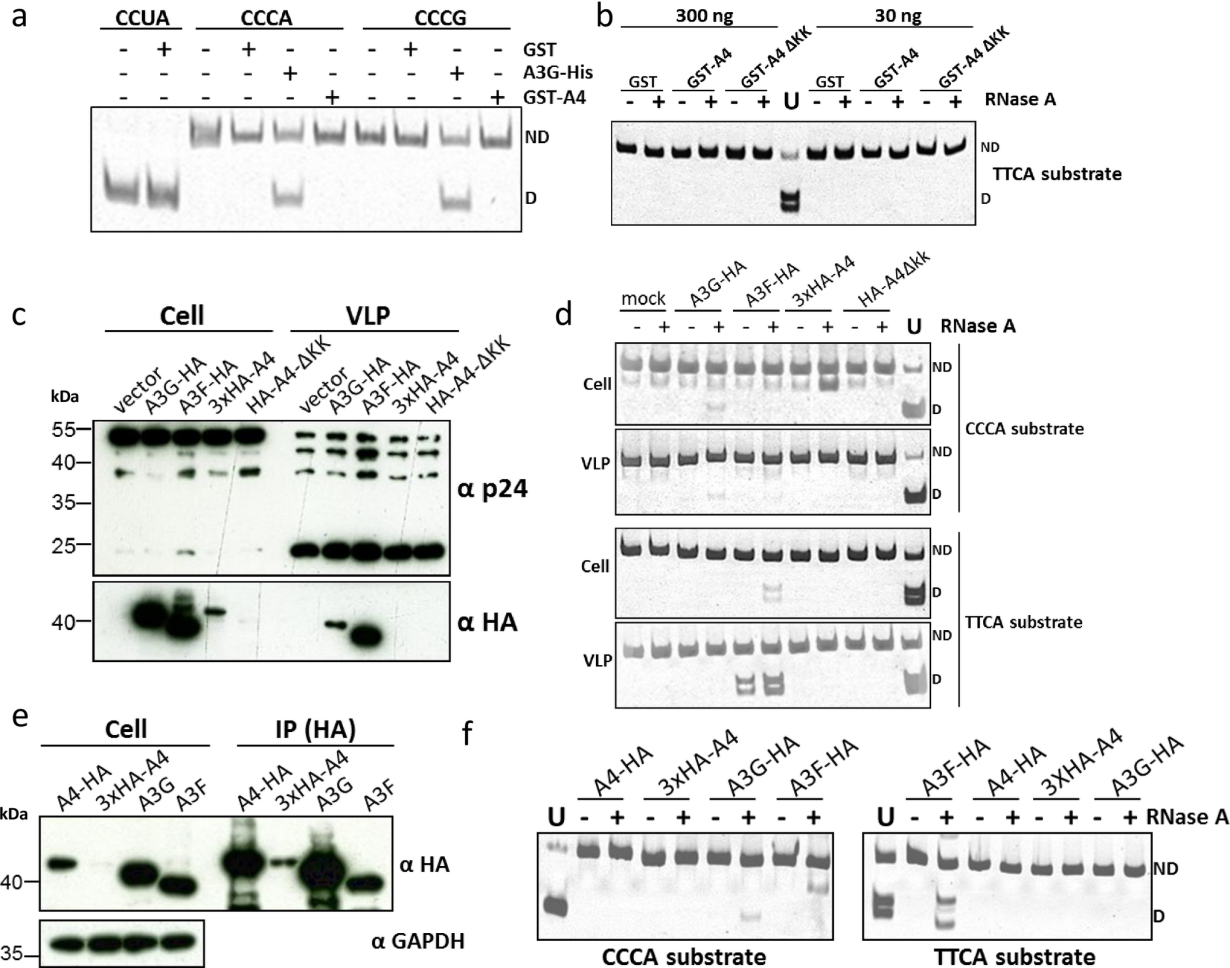
A4 does not deaminate single stranded DNA. (a) Deamination activity of A4 was tested on two different oligonucleotide substrates containing nucleotide sequences CCCA or CCCG. The A3G-His fusion protein was incubated with CCCA and CCCG containing substrates and served as positive control for deamination resulting in 40-bp DNA fragments. Oligonucleotide CCUA served as a marker to denote the deaminated product after Eco147I cleavage; ND: not deaminated; D: deaminated. (b) Deamination experiment using TTCA containing oligonucleotide and GST-purified A4 proteins, RNAse A treatment was included; ND: not deaminated; D: deaminated. (c) Immuno blot analysis of cell lysates and virus lysate of A3G-HA, A3F-HA, 3xHA-A4 and HA-A4-ΔKK expressing cells and HIV virus like particles (VLP), respectively. Anti-HA staining indicates the presence of HA-tagged A3 and A4 proteins, while anti-p24 antibody detects HIV-1 capsid proteins. (d) Deamination assay using transfected 293T cell lysate (from experiment shown in (c)). RNAse A treatment was included; ND: not deaminated; D: deaminated. (e) Immuno blot analysis of cell lysate and immunoprecipitate (IP) fraction of A3 and A4 proteins. (f) Deamination assay using the immunoprecipitated APOBEC proteins (from experiment shown in (e)). RNAse A treatment was included; ND: not deaminated; D: deaminated.

**Fig 11:**
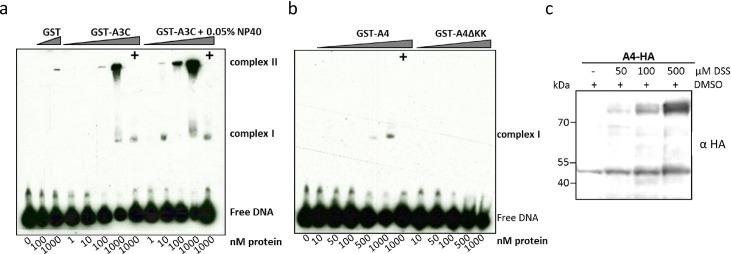
A4 interacts weakly with single-stranded DNA. EMSA with purified, GST-A3C (a), GST-A4 and GST-A4ΔKK (b) performed with 30 nt single stranded target DNA labeled with 3’-labeled with biotin. Indicated amounts of protein (at the bottom of blot) were titrated with 10 nM of DNA. (+) indicates presence of competitor DNA, which is unlabeled 80 nt DNA (200-fold molar excess), as used for deamination assay to demonstrate specific binding of protein to DNA being causative for the shift. For GST-A3C (a) a separate panel was added for reactions containing 0.05% NP-40 detergent. (c) A4-HA crosslinking by DSS. DSS was added to the cleared cell lysates to reach the indicated DSS concentrations. The blot was probed with anti HA antibody to detect monomeric and dimeric forms of A4-HA.

To evaluate the cytidine deaminase activity of A4 directly, we performed *in vitro* cytidine deamination assays as described before [61, 62]. We expressed and purified GST-tagged fusion proteins (GST-A3C, GST-A4, GST-A4ΔKK and free GST) from *E*. *coli* (Fig 9) and used them for activity assays (Fig 10) and DNA binding experiments (Fig 11). In parallel, A3G-His was purified from transfected 293T cells [62] (Fig 9) and used as a positive control for deamination of CCC to CCU. Because the target preference for A4 is not known, we used two different oligonucleotide substrates containing either CCCA/G or TTCA in the central region. If deamination of cytidine to uridine occurred, a 40-nt DNA product is generated after restriction enzyme cleavage and detectable after separation of the digested substrate on a polyacrylamide gel. This method demonstrated cytidine deamination of CCC oligonucleotide substrates by A3G-His protein but not by GST-A4 (Fig 10A and 10B). Since *E*. *coli*-derived GST-proteins might not be optimally folded and may differ in deamination activity or DNA binding due to the GST-tag, we additionally tested APOBEC proteins encapsidated in virions, protein lysates of transfected 293T cells or APOBEC proteins immunoprecipitated from transfected cells (Fig 10C and 10E) for their deamination activity (Fig 10D and 10F). We performed *in vitro* editing experiments with HA-tagged A4, A4-ΔKK, A3F and A3G. In contrast to A3 proteins, A4 were not detected in HIV-1 particles (Fig 10C). Similarly, only minor amounts of 3xHA-A4 were detectable in lysate of transfected 293T cells, but this could be enhanced by immunoprecipitation with HA affinity beads (Fig 10E). We saw deamination of CCC to CCU and TTC to TTU only by A3G and A3F, respectively. A4 did not deaminate in any of the above experiments irrespective of its protein source, tags or target DNA (Fig 10D and 10F).

### A4 weakly interacts with single stranded DNA

For A3 proteins such as A3G, interaction with single-stranded DNA (ss-DNA) and the formation of multiple DNA-protein complexes was shown [61, 63, 64]. We purified GST, GST-A4 and as a control, GST-A3C from *E*. *coli* to characterize whether GST-A4 interacts with ss-DNA. Electrophoretic mobility shift assays (EMSA) were carried out with a biotinylated end-labelled 30 nt DNA oligo (TTCA). GST as a background control protein did not cause any characteristic shift. GST-A3C formed complex I, but a greater proportion shifted on the top of the blot (complex II) at the highest protein concentration (1 μM) (Fig 11A). However, the addition of detergent NP-40 aided to form the stable complex I at 10 nM GST-A3C and complex II at higher protein concentrations, suggesting a strong GST-A3C interaction with DNA. Importantly, the GST moiety did not affect the binding (Fig 11A). GST-A4 did not cause a shift at low protein concentration like A3C or A3G [64], but at the highest amount of protein used (500–1000 nM) a minor proportion of complex I was formed. All the DNA-protein complexes in the EMSA were disrupted by adding the 80 nt unlabeled competitive DNA in 200-fold molar excess. In contrast, GST-A4ΔKK failed to form any complexes (Fig 11B).

Crosslinking DNA-A3G studies previously showed that the deamination activity on ss-DNA was facilitated when A3G formed dimers and tetramers [65]. These observations suggested analyzing the capacity of A4 to form dimers. To demonstrate that A4 protein multimerizes in human cells, cleared cell lysate was incubated with different concentrations of the cross linking reagent disuccinimidyl suberate (DSS). Immunoblot analysis of cross-linked samples dose-dependently revealed the existence of A4 running at the molecular weight expected for dimers, indicating that primary amines which can be crosslinked with DSS are present within the A4 dimerization interface (Fig 11C).

### A4 enhances expression of HIV-1 LTR and other promoters

**Fig 12:**
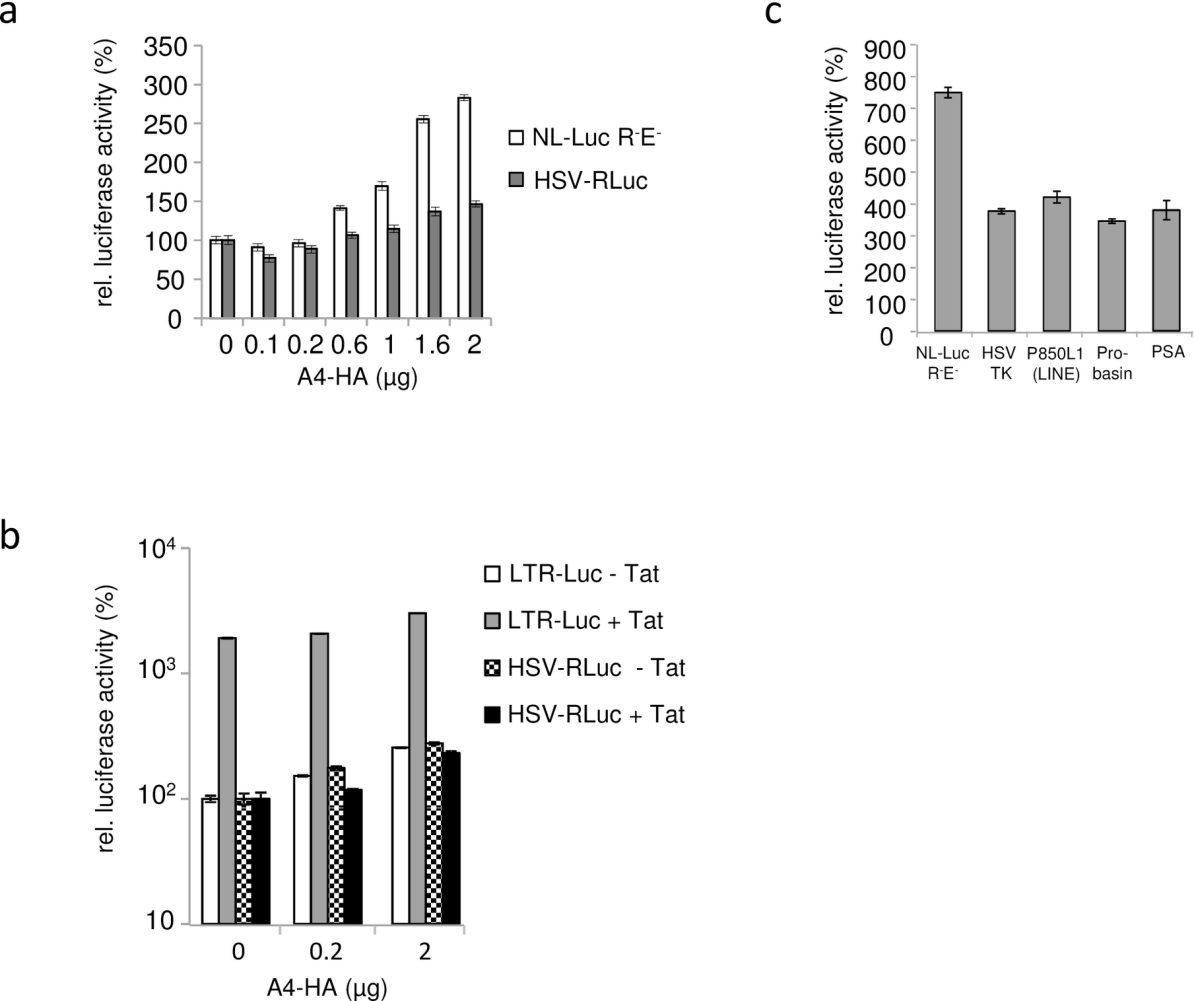
A4 enhances expression of luciferase reporter genes driven by various viral and cellular promoters. (a) Dual luciferase reporter assay was performed two days post co-transfection of NL-Luc R^-^E^-^ and HSV-TK promoter *Renilla* luciferase (HSV-RLuc) with and without A4-HA, relative luciferase activities are shown. (0) indicates transfections in the absence of A4-HA plasmid. A4-HA was transfected in increasing amounts. (b) Relative luciferase activities after co-transfection of LTR-Luc (LTR of HIV-1 driving firefly luciferase) with A4-HA or HSV-TK promoter *Renilla* luciferase (HSV-RLuc) with A4-HA and with and without Tat expression plasmid (c) Luciferase activities driven by various viral (LTR, HSV TK) or cellular promoters (LINE, Probasin, PSA) in presence of the transfected A4-HA expression plasmid, relative to luciferase activity in cells without A4 expression. Total amounts of luciferase expression plasmid and total plasmid DNA was kept constant within all experiments. Error bars indicate standard deviation.

To test whether A4 enhances specifically HIV-1 production, we performed comparative expression analysis of HIV-1 LTR and other viral and cellular promoters with and without A4 in the same cell. To this end, we co-transfected the NL-Luc plasmid expressing the firefly luciferase gene which is located in the *nef* gene together with the Herpes simplex virus (HSV) thymidine kinase (TK) promoter-driven *Renilla* luciferase (HSV-RLuc) reporter plasmid and different amounts of A4-HA expression plasmids. Both luciferases were measured sequentially from single samples. The results revealed an A4 dose-dependent increase in both luciferase activities (up to 2.5-fold for NL-Luc expression and up to 1.5-fold for the HSV driven luciferase) (Fig 12A). To test whether A4 affects HIV expression by acting on the viral LTR, luciferase reporter constructs with the HIV-1 LTR (LTR-Luc, firefly luciferase) and HSV-RLuc were co-transfected with increasing amounts of A4-HA expression plasmid with or without addition of an HIV-1 Tat expression plasmid. As expected, the presence of Tat enhanced the expression of the LTR-Luc construct (by 19-fold) relative to LTR-Luc expression in the absence of the Tat plasmid (Fig 12B). A4 expression in the absence of Tat stimulated the LTR-Luc expression by up to 2.6-fold and by up to 1.6-fold in the presence of Tat. The thymidine kinase promoter of HSV was not sensitive to the presence of Tat. In contrast, A4 enhanced the HSV-RLuc expression by up to 2.8-fold when Tat was not co-transfected. In the next experiment, we tested firefly luciferase expression constructs driven by promoters of HSV-TK, LINE1 (P850 L1), probasin, or prostate-specific antigen (PSA), together with NL-Luc. Co-transfection with 2 μg A4-HA expression plasmid enhanced the luciferase activity of all these constructs from 3.5-fold to 5-fold, whereas HIV-LTR expression was enhanced by 7-fold (Fig 12C). Based on these results, we conclude that A4 might directly or indirectly enhance the transcription of HIV and other promoters.

## Discussion

Herein we report the first study addressing the potential function of the A4 protein in human cells. A4 is the most recently identified and the least studied APOBEC protein [3, 52]. It is more closely related to A1 than to the A3 proteins [3]. Knowledge about the A4 protein is very limited; it is unknown if A4 binds to RNA or DNA or possesses any enzymatic activity, and no biochemical and structural information about A4 is available to date. Our data show for the first time biological activity of A4, which enhances the expression of HIV-1.

As part of this study, we established mammalian expression plasmids for A4 and we also generated bacterially expressed GST-A4 fusion proteins to test for their enzymatic activity. Under experimental conditions that readily detect cytidine deamination by A3G, purified GST-A4 did not carry out any detectable cytidine deamination. We also tested A4 isolated from transfected human cells and similarly found no cytidine deamination activity. These findings are in agreement with the previously reported absence of cytidine deamination of A4 using a cellular mutation assay in bacteria and yeast [52]. In addition, we found that mutating the zinc-coordinating domain of A4 did not abolish the HIV-enhancing activity of A4. Nevertheless, these observations do not mean that A4 is catalytically inactive, A4 may just have different substrate specificity and cytidine deamination may not be the A4 function required for enhancement of HIV expression.

The deamination activity of A3 proteins such as A3G on ss-DNA is facilitated by A3G dimers and tetramers [65]. A4 formed at least dimers, but did weakly bind to ss-DNA only. This weak DNA binding was lost, if the characteristic polylysine stretch (KKKKKGKK) at the C-terminus of A4 was deleted (A4ΔKK), supporting the hypothesis that the net positive charge rendered by polylysines confer some capacity to interact with DNA [66]. Thus, the weak interaction of A4 with ss-DNA may be one reason for the lack of detectable deamination.

We speculated that the polylysine domain would be involved in nuclear localization of A4 and that a C-terminal HA-tag would obstruct this activity, because A4 with an N-terminal HA-tag (HA-A4) localized to both cytoplasm and nucleus of transfected cells, while A4-HA with a C-terminal HA-tag was detected only in the cytoplasm. However, HA-A4ΔKK also localized to both compartments, cytoplasm and nucleus, suggesting that the polylysine domain is not important for nuclear localization. C-terminal stretches of lysines are also found in other proteins unrelated to A4 e.g. in the GTPase KRas (KRAS, K-Ras4B, NP_004976) and FAM133B (NP_001035146). In KRas, the polylysine region (KKKKKKSK) contributes to the interaction of KRas with Ca^2+^/calmodulin and strongly influences its binding to the plasma membrane by electrostatic interactions with the membrane anionic lipids [67, 68]. Whether A4 specifically interacts with membranes or Ca^2+^/calmodulin is not known.

We demonstrated that A4 mRNA is highly expressed in human testis, but is barely detectable in 293T, HeLa, A3.01 T and Jurkat T cell lines. Analysis of protein expression of endogenous A4 was precluded, due to the non-availability of any A4-specific antibody. In light of the sexual transmission of HIV-1 and the possibility of sanctuary sites for HIV-1 in the male genital tract, the origin of seminal HIV-1 is a topic of ongoing discussion [69–76]. Human testicular tissue is described to be susceptible to HIV-1 [69, 72, 77–80] and macaque testis and epididymis are found to be infected by SIV in several studies [70, 73, 76, 81, 82]. Since we do not know whether CD4^+^ cells in testis express A4, we cannot make a statement concerning modulation of HIV infection in testis by A4. A4 also enhanced the expression of firefly luciferase which is controlled by the HIV-1 LTR in a manner similar to that of the unrelated HSV promoter driven *Renilla* luciferase and A4 expression increased the expression of luciferase constructs driven by cellular promoters. However, our results do not clearly demonstrate that A4 is a factor that enhances LTR-mediated transcription. Indeed, it is thus likely that HIV benefits from a broad activity of A4. We hypothesize that A4 creates a cellular/nuclear environment that stimulates for example the expression of HIV-1. A4 may boost expression or activity of a factor important for HIV or reduce the expression or activity of a negative regulator of HIV. It is very well possible that the observed enhancing activity of A4 to HIV is relevant for the expression of cellular promotors and endogenous retroviruses in testis [83]. Future studies investigating the interactome of A4 may help to reveal the A4 pathway and its enhancing activity.

## Material and Methods

### Plasmids

pA4-HA (pA4-3xHA) expresses APOBEC4 (A4, GenBank NM_203454.2) fused to three C-terminal HA-tags; pMH-A4_3xHA (obtained from Matthias Hamdorf) was used to excise A4-3xHA using EcoRI x NotI, cloned into EcoRI / NotI of pcDNA3.1*zeo*(+) (Life Technologies, Darmstadt, Germany). pA4-HA-E95Q was generated by side-directed mutagenesis of the pA4-HA plasmid, the mutation was confirmed by sequencing. pA4 expresses A4 without an epitope tag. pA4 was cloned by PCR (pA4-HA as template) using primers hA4 5’ (5’-CGGATCCCTAGCAATGGAGCCCATATATG) and hA4 3’ (5’-GAATTCTTTATTTCTTCCCTTTCTTCTTCTTC), the PCR product was cloned via BamHI / EcoRI into pcDNA3.1*zeo*(+). HA-A4 (p2xHA-A4): expresses A4 with two N-terminal HA-tags, a pcDNA3.1*zeo*(+)-based plasmid with one N-terminal HA-tag of A4 was generated by PCR using primers HA-hA4 5’ (5’-CGGATCCCTAGCAATGGGATATCCATACGATGTTCCAGATTACGCTGAGCCCATATATGAGGAGTACC) and hA4 3’ (5’-GAATTCTTTATTTCTTCCCTTTCTTCTTCTTC); this plasmid served as template for a second PCR using primers for_2xHA-A4 (5’-CGGGATCCCTAGCAATGGGATATCCATACGATGTTCCAGATTACGCTGGCTATCCATACGATGTTCCAGATTACGCTGGCTATCCATACGATGTTCCAGATTACGCT) and rev rc_hA4 (5’-GCCGGAATTCTTATTTCTTCCCTTTCTTCTTCTTC). The product was cloned via BamHI / EcoRI into pcDNA3.1zeo(+). pGST-A4: A4 with an N-terminal GST-tag in pGEX-6-P1 (GE Healthcare, Munich, Germany), A4 was cloned via BamHI / EcoRI by excising A4 from pA4. Similarly pGST-A4-ΔKK was cloned in pGEX-6-P1 (GE Healthcare, Freiburg, Germany) using forward primer 5’- ATCGGATCCATGGAGCCCATATATGAGGAG and reverse primer 5’-CGGCGAATTCTTATTCATCTGCCTCCTTGCTACT. pMSCV.A4: a murine leukemia virus-based vector to express A4 fused with three C-terminal HA-tags; it was cloned by PCR using template pA4-3xHA and primers A4_fw_RI (5’-TGGAATTCGCCCTTCAGGCGGTACCAGCCTGGAGACAAATTGATG) and A4_rv_RI (5’-TAGAATTCTCAGTTAGCCGGCGTAG)via EcoRI into pMSCV.neo (Clonetech, Takara Bio Europe/SAS, Saint-Germain-en-Laye, France). pLTR-Luc (pGL3-bas-NL43LTR-luc): containing the LTR region of HIV-1 pNL4-3, cloned by PCR of U3, R, and TAR elements using primers NL4-3-U3(+) (5´-CTCGGCAGATCTCTGGAAGGGCTAATTCACTCC) and U3/R/TAR(-) (5´-GCTCGGAAGCTTGGCTTAAGCAGTGGGTTCCCTAG); amplicons were cloned via HindIII and BglII (partial digest) into pGL3-Basic (Promega, Mannheim, Germany). P850 luciferase plasmid with LINE Promotor (P850 L1) [84] and reporter constructs with androgen responsive promotors probasin (pGL3Eprob) and PSA (pPSA61-luc) [85] were kindly provided by Wolfgang A. Schulz. APOBEC3G (A3G)-HA expression construct was kindly provided by Nathaniel R. Landau [17]. His-tagged huA3G (A3G-Myc-6His) has been described previously [86]. APOBEC3A (A3A)-HA expression plasmid was obtained from Bryan R. Cullen [87]. pTat (pBS-KRSPA-Tat NL4-3), expressing Tat protein of HIV-1 NL4-3 was a gift of Heide Muckenfuss and Egbert Flory. For cloning of pTat, both Tat exons were amplified and fused by PCR using pNL4-3 [56] as template, and cloned into XhoI / SpeI of pBS-kRSPA [88]. pHSV-RLuc (pRG-TK, Promega), *Renilla reniformes* luciferase expressed by the *Herpes simplex virus type 1* thymidine kinase promoter.

### Cells, transfections and infections

HOS (ATCC CRL-1543), HOS.CD4.CCR5 [60], HeLa (ATCC CCL-2), TZM-bl [59] and 293T (ATCC CRL-3216) cells, were maintained in Dulbecco’s modified Eagle’s medium complete (PAN-Biotech, Aidenbach, Germany); A3.01 T cells [89] and Jurkat T cells clone E61 (ATCC TIB152) were cultured in Roswell Park Memorial Institute (RPMI) 1640 medium (PAN-Biotech) supplemented with 10% FBS, 0.29 mg/ml L-glutamine, and 100 U/ml penicillin/streptomycin at 37°C in a humidified atmosphere of 5% CO_2_. Plasmid transfections into 293T cells were performed with Lipofectamine LTX (Life technologies). HIV-1 and reporter lentiviruses were generated by transfection in 6-well plates with 200 ng pNL.Luc R^-^ E^-^ (pNL4-3.Luc.R^–^.E^–^) [57] plus 50 ng vesicular stomatitis virus G glycoprotein (VSV-G) expression plasmid pMD.G or with 1 μg pNL4-3 [56] and different amounts of A4 expression plasmid. Total transfected plasmid DNA was maintained by adding appropriate amounts of pcDNA3.1zeo(+) plasmid DNA where needed. To produce NL4.3 with the *env* BaL, pNL-BaL [58] was transfected in 293T cells. Reverse-transcriptase (RT) activity was determined using the Cavidi HS kit Lenti RT (Cavidi Tech, Uppsala, Sweden). For infectivity assays, 4x10^3^ HOS cells were transduced in 96-well plates in triplicate with a virus amount equivalent to 10 pg of RT for HIV. Three days post infection, luciferase activity was measured using the Steadylite HTS kit (PerkinElmer, Rodgau, Germany). To quantify firefly and *Renilla* luciferase in the same cell lysate, the dual-Luciferase reporter assay (Promega) was applied. All luciferase assays in transfected cells were performed two days post transfection. To generate stable A4-expressing HOS.CD4.CCR5.A4 and control HOS.CD4.CCR5.neo cells, pMSCV.A4 or pMSCV.neo plasmid was co-transfected together with pHIT60 [90] and pMD.G for generation of murine leukemia viral vector particles. Vector particles were used to transduce HOS.CD4.CCR5 cells; G418 resistant cells were pooled and characterized for CD4 and CCR5 receptor and A4 expression. Spreading virus replication with NL-BaL was quantified over 20 days infecting HOS.CD4.CCR5.neo or HOS.CD4.CCR5.A4 cells using a multiplicity of infection of 0.01 and testing the culture supernatants with the HIV reporter cell line TZM-bl [59]. Transfection efficiency was monitored by cotransfection of 100 ng Monster Green fluorescent protein expression plasmid hMGFP (Promega).

### Immunoblot analysis

Cells were lysed in radioimmunoprecipitation assay buffer (RIPA, (25 mM Tris (pH 8.0), 137 mM NaCl, 1% glycerol, 0.1% SDS, 0.5% Na-deoxycholat, 1% Nonidet P-40, 2 mM EDTA, and protease inhibitor cocktail set III [Calbiochem, Darmstadt, Germany].) buffer, 20 min on ice. Lysates were clarified by centrifugation (10 min, 300 g, 4°C). Samples were boiled in NuPAGE SDS Sample Buffer and NuPAGE Sample Reducing Agent (Life technologies) and subjected to SDS-PAGE followed by transfer to a PVDF membrane. A3G and A4 Proteins were detected using an anti-HA antibody (Ab) (1:10^4^ dilution, MMS-101P; Covance, BioLegend, Fell, Germany), HIV-1 p24 Gag was detected applying HIV-1 p24 monoclonal Ab (1:250 dilution, AG3.0, NIH AIDS REAGENTS, Germantown, USA) [91]. Cell lysates were probed with α-tubulin Ab (1:10^4^ dilution, B5-1-2; Sigma-Aldrich, Munich, Germany) and virions with α-p24 monoclonal Ab 183-H12-5C. Vif protein was detected with HIV-1 Vif monoclonal antibody (1:5x10^3^ dilution, #319, NIH AIDS REAGENTS) [92]. Secondary Abs.: anti-mouse (NA931V) and anti-rabbit (NA934V) horseradish peroxidase (1:10^4^ dilution, GE Healthcare). Signals were visualized using ECL reagent (GE Healthcare).

### Chemical cross linking

293T cells were transfected with pA4-HA and lysed two days after transfection with RIPA buffer. Soluble fraction was clarified by centrifugation at 13,000 rpm and 4°C. To chemically cross link the amines of the protein, the lysate was treated with various concentrations of disuccinimidyl suberate (DSS) (Thermo Scientific, Braunschweig, Germany) dissolved in DMSO to make a final concentration of 50, 100 and 500 μM and the reaction mixture was incubated for 20 min on ice. To quench the reaction, 20 mM of Tris (final concentration) was added and lysates were subjected to immunoblot analysis without addition of reducing reagent. The presence of A4 monomers and dimers were detected by anti HA antibody.

### PCR

Total RNA was isolated using RNeasy mini kit (Qiagen, Hilden, Germany). Human testis RNA (DNase free, HR-401) was obtained from Zyagen (San Diego, USA). RNA was reverse transcribed with QuantiTect Reverse Transcription (Qiagen). Semi-quantitative PCR analyses of A4 mRNA: The A4 fragments were amplified from cDNA by Dream-*Taq* polymerase (Thermo Scientific) and the primers Origene_for (5‘-CAAGCCTGGAGACAAATTGATGG) x Origene_rev (5‘-GCAATCGAGAGAGAAGCTTAGCC). As a control, β-2-microglobulin cDNA was amplified in the same PCR reaction applying primer β-2-Mikroglobulin A_for (5’-CTCGCTCCGTGGCCTTAGCTGTGCTCGCGC) x β-2-Mikroglobulin A_rev (5’-TAACTTATGCACGCTTAACTATC): Initial denaturation at 95°C for 5 min followed by 39 cycles of 95°C for 1 min, 56°C for 1 min, 72°C for 1 min and final extension 72°C for 15 min. Water instead of template served as a background control and a plasmid coding for A4 cDNA (pA4 cDNA) served as a positive control. The identity of the PCR fragments was confirmed by cloning and sequencing. Quantitative real-time PCR analyses of A4 mRNA: The A4 fragments were amplified from cDNA using SYBR green PCR Master Mix (Applied Biosystems, Warrington, United Kingdom) with an Applied Biosystems 7500 Real-Time PCR system (Applied Biosystems, Foster City, CA) and primers: A4_909_for (5’-ACCAATGCATATGGGCCAAA) x A4_906_rev rc (5’-GTGCCTTACGATATTCCTGGGT). After initial incubations at 50°C for 2 min and 95°C for 10 min, 40 cycles of amplification were carried out for 15 s at 95°C, followed by 1 min at 60°C. The amplification product was normalized to that of HPRT1 using PCR Primers HPRT1_for (5’-GCTTTCCTTGGTCAGGCAGT) x HPRT1_rev rc (5’-GCTTGCGACCTTGACCATCT).

### Purification of A3 and A4 proteins from *E*. *coli* and 293T cells

A3G-His was expressed in 293T cells and purified by immobilized metal affinity chromatography (IMAC) using Ni-nitrilotriacetic acid (Ni-NTA) agarose (Life Technologies) as described [62]. GST-A3C, GST-A4, GST-A4ΔKK and GST proteins were overexpressed in *E*. *coli* Rosetta (DE3) cells (Millipore, Merck Chemicals, Darmstadt, Germany) and purified by affinity chromatography using Glutathione Sepharose 4B beads (GE healthcare). After the growth of transformants containing pGEX4T2-GST-A4 until 0.6 OD_600_, cells were induced with 1 mM isopropyl-beta-D-thiogalactopyranoside (IPTG) and 1 μM ZnSO_4_ and cultured at 18°C overnight. A4 harboring cells were washed with PBS and lysed with 1X Bug buster protein extraction reagent (Millipore) containing 50 mM Tris (pH 7.0), 10% glycerol, 1 M NaCl and 5 mM 2-mercaptoethanol (2-ME), clarified by centrifugation (14,800 rpm for 20 min at 4°C) and the soluble fraction was mixed with glutathione sepharose beads. After 3 h incubation at 4°C in end-over-end rotation, the beads were washed twice with wash buffer containing 50 mM Tris (pH 8.0), 5 mM 2-ME, 10% glycerol and 500 mM NaCl. The bound GST-A4 protein was eluted with wash buffer containing 20 mM reduced glutathione. Purified protein concentration was determined spectrophotometrically by measuring the *A*_280_, using their (theoretical) extinction coefficient and molecular mass.

### *In vitro* DNA cytidine deamination assay

Deamination reactions were performed as described [61, 62] in a 10 μL reaction volume containing 25 mM Tris pH 7.0, and 100 fmol single stranded DNA substrate (CCCA: 5'-GGATTGGTTGGTTATTTGTTTAAGGAAGGTGGATTAAAGGCCCAAGAAGGTGATGGAAGTTATGTTTGGTAGATTGATGG; CCCG: 5'-GGATTGGTTGGTTATTTGTTTAAGGAAGGTGGATTAAAGGCCCGAAGAAGGTGATGGAAGTTATGTTTGGTAGATTGATGG and TTCA: 5’- GGATTGGTTGGTTATTTGTATAAGGAAGGTGGATTGAAGGTTCAAGAAGGTGATGGAAGTTATGTTTGGTAGATTGATGG). Samples were splitted into two halves; in one half 50 μg/ml RNAse A (Thermo Scientific) was added. Reactions were incubated for at least 1 h at 37°C and the reaction was terminated by boiling at 95°C for 5 min. One fmol of the reaction mixture was used for PCR amplification (Dream *Taq* polymerase (Thermo Scientific) 95°C for 3 min, followed by 19 cycles of 61°C for 30 sec and 94°C for 30 sec) and the primers forward 5'-GGATTGGTTGGTTATTTGTTTAAGGA, reverse 5'-CCATCAATCTACCAAACATAACTTCCA used to amplify CCC(A/G) substrate, forward primer 5’-GGATTGGTTGGTTATTTGTATAAGGA with the above reverse primer used for TTCA. PCR products of CCC(A/G) and TTCA were digested with Eco147I (StuI) (Thermo Scientific) and MseI (NEB, Frankfurt/Main, Germany), respectively, resolved on 15% PAGE, stained with ethidium bromide (5 μg/ml). As a positive control substrate oligonucleotides with CCUA and TTUA instead of respective CCCA and TTCA were used to control the restriction enzyme digestion.

APOBEC incorporation into HIV-1: HIV-1 vectors were produced with 250 ng A3 plasmids and 1000 ng A4 constructs. 48 h later virion containing supernatants were concentrated by layering on 20% sucrose cushion and centrifuged for 4 h at 14,800 rpm. Viral particles were re-suspended in mild lysis buffer (50 mM Tris (pH 8), 1 mM PMSF, 10% glycerol, 0.8% NP-40, 150 mM NaCl and 1X complete protease inhibitor) and used as input for the *in vitro* deamination assay.

Deamination assay using immunoprecipitated protein from 293T cells: 293T cells were transfected with expression plasmids encoding A4-HA, 3xHA-A4, A3G-HA or A3F-HA. Cells were lysed 48 h post transfection with mild lysis buffer (50 mM Tris (pH 8.0), 1 mM PMSF, 10% glycerol, 0.8% NP-40, 150 mM NaCl and protease inhibitor (protease inhibitor cocktail set III, Calbiochem). HA-tagged proteins were immunoprecipitated using 20 μl of anti-HA Affinity Matrix Beads (Roche Diagnostics, Mannheim, Germany) by slowly rotating the lysate bead mixture for 2 h at 4°C. One third of the beads were used for deamination assay and the remaining was used for immunoblot analysis.

### Electrophoretic mobility shift assay (EMSA) with GST-A3C and GST-A4

EMSA method is adapted from [63, 64]. Proteins were produced as described above, kept in protein buffer (final concentration 50 mM Tris (pH 8.0), 50 mM NaCl, and 10% glycerol). 10 mM 3’ biotinylated DNA (30-TTC-Bio-TEG purchased from Eurofins Genomics, Ebersberg Germany) was mixed with 10 mM Tris (pH 7.5), 100 mM KCl, 10 mM MgCl_2_, 1 mM DTT, 2% glycerol, and desired amount of recombinant proteins in a 10 μl reaction mixture, and incubated at 25°C for 30 min. The protein-DNA complex was resolved on a 5% native PAGE gel on ice, and then transferred onto nylon membrane (Amersham Hybond-XL, GE healthcare) by southern blot. After transfer, the molecules on the membrane were crosslinked by UV-radiation using a transilluminator at 312 nm for 15 min. Chemiluminescent detection of biotinylated DNA was carried out according to the manufacturer’s instruction (Thermo scientific).

### Confocal microscopy

1 x 10^5^ HeLa cells grown on coverslips (Marienfeld, Lauda Königshofen, Germany) were transfected with 500 ng A4 expression plasmids by applying Lipofectamine LTX transfection reagent. At day two post transfection, cells were fixed in 4% paraformaldehyde in PBS for 30 min, permeabilized in 0.1% Triton X-100 in PBS for 45 min, incubated in blocking solution (10% donkey antiserum (Sigma-Aldrich) in PBS) for 1 h, and treated with anti-HA Ab (MMS-101P; Covance) in blocking solution for 1 h. Donkey anti-mouse Alexa Fluor 488 (Life Technologies) was used as secondary Ab in a 1:300 dilution in blocking solution for 1 h. Finally, nuclei were stained using DAPI (4’, 6’-diamidino-2-phenylindole; 1:1000 in PBS) (Merck Millipore, Darmstadt, Germany) for 5 min. Coverslips were mounted on glass microscope slide (Marienfeld) using Fluorescent Mounting Medium (DAKO, Hamburg, Germany). The images were captured by using a 63x objective on a Zeiss LSM 510 Meta laser scanning confocal microscope. x-z optical sections were acquired from 0.28 μm layers.

### Statistics

Evaluation of RT or reporter activity data was performed by means of a multifactorial analysis of variance (ANOVA) with fixed factor *plasmid ratio*. Additionally a random factor *day* was included, if more than one determination were obtained from one day in order to model day-to-day variability. The statistical analysis was performed with SAS/STAT software, version 9.3, SAS System for Windows (Cary, USA).
